# Exploration of service centres for older persons in the Eastern Cape province, South Africa

**DOI:** 10.4102/sajp.v78i1.1567

**Published:** 2022-01-28

**Authors:** Ntsikelelo Pefile, Bomkazi Fodo, Seyi Amosun

**Affiliations:** 1 Department of Health and Rehabilitation Sciences, Division of Physiotherapy, Stellenbosch University, Cape Town, South Africa; 2 School of Health Sciences, Division of Physiotherapy, University of KwaZulu-Natal, Durban, South Africa; 3 Department of Health and Rehabilitation Sciences, Division of Physiotherapy, University of Cape Town, Rondebosch, South Africa

**Keywords:** population ageing, active ageing, service centres for older persons, WHO active-ageing framework, Department of Social Development

## Abstract

**Background:**

Service centres for older persons were set up in South Africa to implement programmes relating to the six determinants of the World Health Organization’s (WHO) active ageing framework. The framework highlights the value of being physically active to prevent functional decline.

**Objective:**

Our aim was to explore the characteristics of these centres and their members in the Eastern Cape province.

**Method:**

An exploratory, descriptive, cross-sectional design was utilised to explore the characteristics of 25 centres and the profiles of their managers and 275 members of these centres.

**Results:**

The managers had no formal training. Health and social care system requirements were important in the province, but access to healthcare services was minimal, and managers were concerned about the physical environment. Over 50% of the centres provided meals (72%), social support services (60%), dance and aerobics (56%), blood glucose testing (52%) and guardianship for members without families (52%). The members reported multiple morbidities, including hypertension (59%), diabetes (16%) and arthritis (10.5%). Few members used tobacco (*n* = 20) and alcohol (*n* = 27), but most (*n* = 213) were afraid of falling although they went about their daily activities with minimal difficulties. Members were satisfied with their lives (*n* = 231).

**Conclusion:**

The centres provided platforms to enable the fulfilment of some of the goals of the WHO’s active-ageing framework, but a comprehensive exploration of the centres and the members is needed.

**Clinical implications:**

Physiotherapy, as part of interdisciplinary intervention, will promote the physical health of the members of the centres.

## Introduction

The United Nations (2017) has projected that, globally, the number of older persons, aged 60 years and older, will be about 2 billion in 2050. In 2015, life expectancy after 60 years, which is the average number of years that a person of that age can be expected to live if age-specific mortality levels remain constant, ranged from 13 years in Sierra Leone, a low-income country in Africa, to 26 years in Japan, a high-income country and an increasing trend was observed (Help Age International 2015). The World Health Organization (WHO) considered these developments as pointers to the success of public health policies whilst challenging society to make the necessary adaptations to enable older persons to maximise their ‘health and functional capacity’ as well as their ‘social participation and security’ (WHO 2002a). However, the projected global increases, coupled with a high disease burden, raise public health challenges. Multi-morbidities from long-term illnesses are prevalent amongst older persons and associated risk factors include smoking, alcohol consumption, living in rural areas, low levels of schooling, polypharmacy and a negative self-perception of health (Ayodede et al. 2017). In addition to the routine ageing processes, the consequences of multi-morbidities include a more significant risk of death and functional decline, negatively impacting life expectancy (Gyasi & Phillips 2018; Hosseinpoor et al. 2012; Ofori-Asenso et al. 2019). Functional decline is often unrecognised until it manifests clinically and becomes associated with increasing frailty. If left undetected, it can result in disability and increased healthcare costs, whilst early detection could lead to interventions that may slow down, or stop, the decline. The concept of community-based, non-residential day centres for older persons (also referred to as senior citizens’ centres) was first introduced in New York City in the United States of America (USA) in 1943 to address these challenges (Wick 2012). Similar centres have also been set up in South Africa, named Service Centres for Older Persons (Republic of South Africa 2006).

In 2002, the WHO introduced the global active ageing policy framework, describing active ageing as the process of optimising opportunities for health, participation and security to enhance the quality of life of older persons by focusing on three major goals, namely older persons and development, advancing health and well-being into old age and ensuring an enabling and supportive environment (WHO 2002b). The framework identified six determinants of active ageing: behaviour, personal factors, health and social services, physical environment, social environment and economic determinants. Each determinant has a list of focus activities aimed at achieving the three goals of active ageing. It was intended that the framework would help WHO member countries to plan and guide policies and programmes that would respond to the needs of older persons. Therefore, member countries were expected to have evaluated how the 2002 framework applies to their countries in the first half of the 21st century. The importance of physical activity as a means of limiting functional decline was raised in the framework (Abdullah & Wolbring 2013; WHO 2002b). A person’s level of functioning was conceptualised as a dynamic interaction between an individual and the environmental and personal factors in an individual’s context (WHO 2001). The World Confederation for Physical Therapy (2015), therefore, encouraged physiotherapists to work with legislative and regulatory bodies and service providers to incorporate principles of health promotion interventions into their national programmes for older persons. Physiotherapy has, therefore, been identified as a key part of an integrated and coordinated interdisciplinary team that focuses on enhancing the performance of daily activities considered as necessary by older persons (Abdi et al. 2019; Amosun & Doyle-Baker 2019; Amosun & Harris 2018; Bezner 2015; Siemonsma et al. 2018; Tuntland et al. 2015).

Studies relating to the relevance of the active ageing policy framework have been conducted in some countries, such as Indonesia (Arifin, Braun & Hogervorst 2012), Canada (Bélanger et al. 2017), Europe and Latin America (Bowling 2006; Fernández-Ballesteros et al. 2010; Paúl, Ribeiro & Teixeira 2012) and Thailand (Chansarn 2012). The study by Bélanger et al. (2017) reported some limitations in using the WHO framework but concluded that the framework was still useful to highlight what remained to be improved about the health, social participation and social and economic security of older persons. Unfortunately, there is a paucity of evidence from African countries on the relevance of the WHO’s active-ageing policy framework in the care of older persons (Mapoma 2014). The applicability of the 2002 framework to service centres for older persons in South Africa is therefore worth exploring.

South Africa, a multi-ethnic nation, has the highest level of inequality in the world (Rowe & Moodley 2013). The country additionally has a quadruple burden of diseases (Mayosi & Benatar 2014), namely human immunodeficiency virus (HIV) and acquired immunodeficiency syndrome (AIDS), with a synergistic relationship with tuberculosis (TB); maternal and child morbidity and mortality; non-communicable diseases driven mainly by risk factors related to lifestyle and violence, injuries and trauma. However, integrated primary healthcare services have become more accessible to address the burden of disease, and the use of community health workers (CHWs) in this setting has significantly improved access in peri-urban and rural areas (Austin-Evelyn et al. 2017; Laurenzi et al. 2021). In this context, the population of older persons has gradually increased from 3.28 million in a population of 45 million in 2001 to an estimated 5.4 million in a population of 59.62 million in 2020 (Statistics South Africa 2020). The average life expectancy also increased from 55.09 to 64.12 years within the same period. Life expectancy at 60 years was 16 years in 2015 (Help Age International 2015). In 1995, 385 registered service centres for older persons in South Africa were set up and funded by government and non-government organisations (Van Rensburg & Strydom 2012). In 2011, there was anecdotal evidence that the number had increased to over 1350 centres, with a membership of over 75 000 older persons.

As part of the post-apartheid transformation processes to correct the past’s inequities, the government developed the National Development Plan (NDP) 2030 (South African Government 2011). One of the NDP goals, managed by the National Department of Health (DOH), was to improve the health and well-being of the population across all population groups, thereby raising the average life expectancy to at least 70 years by 2030. The guideline for national and provincial government policies to achieve the NDP goal, namely the *Older Persons Act* of South Africa 2006, managed by the National Department of Social Development (DSD) (Republic of South Africa 2006), was informed by the WHO framework on active ageing (WHO 2002b). In anticipation of the increase in life expectancy and the accompanying increase in the numbers in future generations of older persons in South Africa, it was necessary to explore factors that would contribute to the attainment of the 70 years set as a goal in the NDP 2030, so that these factors could inform the services available at the service centres for older persons. Abdi et al. (2019) had recommended that to develop effective services to address the needs of older persons, it was essential first to understand their care and support needs. Regrettably, caring for older persons in the country still focuses more on the sick than the well person (Amosun 2014; Frost et al. 2015; Naidoo et al. 2019; Phaswana-Mafuya et al. 2013; Ramklass et al. 2010; Tanyi & Pelser 2019). This deficit-focused approach limits the exploration of the individual strengths of older persons.

In 2015, a research grant was obtained by the third author from the South African Medical Research Council to carry out a preliminary exploration of the characteristics of the centres in three of the nine provinces – Eastern Cape, the North-West and Western Cape (Amosun & Harris 2018). Socio-economically, the Western Cape is considered as one of the most developed provinces and the Eastern Cape and North-West as two of the poorest provinces (Ned, Cloete & Mji 2017; Statistics South Africa 2020). Ralston (2018) hypothesised that the community contexts of older persons in South Africa impact their ageing process. The Eastern Cape province is under-resourced and rural and faces considerable barriers to access general services (Ned et al. 2017). The community, in a province, observed for its summer and winter rainfall (Zengeni, Kakembo & Nkongolo 2016), is characterised by poor infrastructure and scattered villages. Most households does not have access to potable water, electricity or proper sanitation. Public transport is limited, and the substandard gravel roads make travelling difficult, long and sometimes impossible, whenever the weather is inclement. Primary care clinics managed by nurses are available, and it is not uncommon that no rehabilitation therapists, such as physiotherapists, are assigned to these clinics. Statistics from the clinics recorded health problems typical of a rural context with deep-rooted poverty: high levels of TB, HIV and AIDS, teenage pregnancy and chronic diseases of lifestyle (mainly hypertension, arthritis, diabetes and mental health disorders including substance-induced psychosis). The province’s estimated population was over 6.73 million in 2020, with older persons making up 11.45% (Statistics South Africa 2020). The life expectancy at 60 years was 16.3 years in 2005, which had increased to 18.0 years by 2012 (Msemburi et al. 2016). It was estimated that approximately 5.5% of the provincial population was 70 years and older in 2020, the highest in the country (Statistics South Africa 2020).

This article reports the outcomes of a preliminary exploration of the characteristics of the organisational structures and members of the service centres for older persons in the Eastern Cape province. In view of the recommendations of the World Confederation for Physical Therapy (2015), it is hoped that these outcomes will provide data from which the contributions of physiotherapy in the care of older persons can be understood.

## Methods

The methodology for this study has been described in an earlier publication (Amosun & Harris 2018). This two-phased study utilised an exploratory, cross-sectional, descriptive, quantitative research design. The target population consisted of all service centres for older persons in the Eastern Cape province that were registered with the provincial DSD in 2017 (*n* = 239) (Table 1). Assistance was sought and obtained from the DSD to inform the managers of the centres about the proposed study. Centres not registered with the DSD were excluded.

**TABLE 1:** Service centres for older persons in the Eastern Cape province.

Districts	Number of registered centres†	Number of registered members†	Number of centres selected	Number of centres involved in study
Alfred Nzo	24	859	9	3
Amathole	39	Unknown	14	6
Sarah Baartman	30	1488	11	5
Buffalo City	30	Unknown	11	0
Chris Hani	32	1398	12	0
Joe Gqabi	24	872	9	0
Nelson Mandela Bay	16	1874	6	4
OR Tambo	44	1628	16	7

**Total**	**239**	**12 327**	**88**	**25**

*Source*: 2017 database of provincial DSD

† , Population of older persons = 569 240, source information from Statistics South Africa estimated mid-year population in 2017.

The sample size of 88 centres was calculated using a population proportion-sample size calculator (https://select-statistics.co.uk/calculators/sample-size-calculator-population-proportion/), assuming a 15% sample proportion, a confidence level of 90% and a 5% margin of error. The number of centres selected from each district was proportional to the number of registered centres in that district. The managers of the selected centres were expected to communicate in English or Xhosa. Regrettably, because of email or cell phone communication challenges, it was difficult to communicate with the managers to obtain their prior consent to take part in the survey. Similarly, the numbers of members in some centres were unavailable from the provincial DSD database. Thus, a convenient sample of the members aged 60 years or older who attended the selected centres on the day of data collection, consented to participate in our study and communicated in either English, Afrikaans or Xhosa was selected.

### Measurement tools

A questionnaire that had been utilised in studying 500 senior centres in the USA (Casteel, Nocera & Runyan 2013) was adapted to explore the participating centres’ organisational characteristics. The adaptation involved the addition of new questions and a complete change or minor alterations to questions to ensure relevance to the South African context. Experts in gerontology validated the content validity of the adapted instrument, which included 28 closed-ended questions designed to capture the centre’s geographical location, the organisation to which the centre was affiliated, its operational budget, demographic information of the managers and training they had received to manage the centres, membership enrolment, managers’ perceptions of members’ needs and specific services available. Two additional open-ended questions specifically asked the managers to describe the training they think they should receive and factors that influenced the types of programmes their centres offered.

To explore the characteristics of the members of the centres, three instruments were administered. The first instrument was a self-developed questionnaire with questions from the WHO study on global ageing and adult health (He, Muenchrath & Kowal 2012) and the Fall Prevention and Education Programme developed by a Department of Physical Therapy in the United States of America (Murphy & Lowe 2013), which also included questions relating to the health promotion needs of older persons (Bertera 1999). The instrument posed questions relating to demographic information, self-reported social support, leisure activities, health history and medication use. Experts in gerontology also verified the content validity of the 40-item instrument consisting of closed-ended questions, except for the last one, which asked participants to describe their plans for the immediate future. The remaining two instruments were standardised questionnaires (Federici, Meloni & Presti 2009; Gureje et al. 2008), namely the WHO Quality of Life – Brief Version (WHOQOL-BREF) and the WHO Disability Assessment Schedule (WHODAS II).

### Procedure

Fieldworkers were employed and trained to serve as research assistants and translators. We facilitated the training, which involved taking the fieldworkers through all the questionnaires to ensure uniformity and accuracy of data collection procedures. The training emphasised the need to refrain from being judgemental or critical of the responses of the participants. The fieldworkers signed a confidentiality clause for handling the participants’ responses. After obtaining signed informed consent from each manager, one of our research team members was solely responsible for data collection in phase one through face-to-face interviews of the managers. In phase two, after obtaining signed informed consent from members of the centres, we administered the three survey instruments, also through face-to-face interviews.

Data were collected from September 2017 to February 2018. Most of the centres selected were in rural areas, which were extremely remote from the main road, and the roads were not easily accessible. So, it was not easy to access the targeted centres whenever it rained. This led to immediate changes in selecting available and accessible centres in the same local areas as the targeted centres, to meet the necessary sample size. The data for phases one and two were collected on the same day to maximise time use.

Unfortunately, the research team was involved in a significant motor vehicle accident on 21 February 2018, following Nelson Mandela Bay’s data collection. This was reported to the police. The team received medical care and were advised to take some time off to recover from the trauma. All attempts to continue with the data collection process were unsuccessful as the fieldworkers terminated their participation because of their cultural beliefs. After due consultation with the wider research team, preliminary analyses of the data collected indicated a common trend in the findings in the centres visited in five of the eight districts in the province, so data collection was stopped.

### Data analysis

Following data collection, our team collected and collated the questionnaires from the fieldworkers and reviewed them for accuracy and completeness. Data were then captured by a Microsoft Access (2010 version) database and re-checked against the raw data to avoid data entry error. Where data relating to a centre or manager was missing, we contacted the specific site. Our team consulted with the fieldworkers to address incorrectly entered data relating to the members. After that, the data were cleaned before exporting to an Excel spreadsheet for further analyses. In the absence of previous studies, the survey followed an exploratory methodology rather than a hypothesis-driven approach. All data collected were analysed descriptively, using the Statistical Package for the Social Sciences (SPSS) version 5.1 software for quantitative data, and outcomes were expressed in frequencies and percentages.

### Ethical considerations

Ethical approval for the larger study was obtained from the Human Research Ethics Committee of the University of Cape Town (HREC reference number: 315/2014). The study’s ethics approval that focused on the Eastern Cape province was later obtained from the Biomedical Research Ethics Committee of the University of KwaZulu-Natal (reference number: BE627/16).

## Results

The 25 service centers were in rural (*n* = 13), peri-urban (*n* = 10) and urban (*n* = 2) areas. They had existed for an average of 14.9 ± 8.1 years (3–20 years) and had been registered with the provincial DSD for an average of 10.4 ± 4.9 years (4–14 years). The managers (*n* = 25) reported that 22 centres had membership enrolment of up to 100 members and only two centres had up to 300 members. Only one centre was affiliated to the provincial DOH, and there were nurses on-site during the survey at this centre. Most centre managers (64%) were in full-time employment, whilst the rest were employed part-time. The managers had primary school (*n* = 4), high school (*n* = 14), college (*n* = 5) and university (*n* = 2) education. The training received by 56% of the managers (*n* = 25) was unstructured but included conflict management, financial management and home-based care. The most important needs of older persons that the managers perceived should be addressed in the centres were poverty alleviation through farming or gardening (28%), socialisation (24%) and health promotion (12%). The managers proposed that their training programmes’ focus should include learning about the behavioural patterns of older persons, the handling of older persons and counselling. To run the centres, the managers reported that the provincial DSD provided R200.00 per capita per person, which was allocated as follows: R90.00 for administration, R30.00 for recreation, R70.00 for nutrition and R10.00 for nursing. The managers perceived that this allocation was not sufficient to meet the needs, and, consequently, expenditure was according to what they considered a priority. Attempts to generate income were unsuccessful. Twelve managers complained that the environments in which their centres were located made accessibility difficult for older persons, and only three centres had transport for their members. The services available in the centres were provided in the local languages of the members, and these services are listed in Table 2.

**TABLE 2:** Services available at the service centres for older persons (*n* = 25).

Available programmes	*n*	%
Meals	18	72
Social support services	15	60
Dance and aerobics	14	56
Blood glucose testing	13	52
Guardianship for members without families	13	52
Yoga, pilates or stretch exercises	12	48
Swimming or water aerobics	12	48
Arts and crafts	11	44
Safety programme in medication use	10	40
Blood pressure monitoring	9	36
Social activities	9	36
Cholesterol screening	8	32
Walking programmes	8	32
Outreach programmes	8	32
Falls screening	7	28
Balance or strength training	7	28
Chronic disease management	5	20
Education programmes for members	5	20
Vision or hearing screening	4	16
Home safety programme	4	16
Transport	3	12
Education programmes for caregivers	2	8
Information on housing	1	4
Others	3	12

A total of 275 members, with an average age of 70.4 ± 14.6 years and predominantly women (*n* = 239), were surveyed. Whilst 35 members had no formal education, the rest had a primary school (38.2%), high school (41.8%) and tertiary (7.3%) education. Most members (47.3%) were widowed, whilst 27.3% were married and lived with their spouses. Most members (67.6%) lived with their children or extended family, whilst only 10 lived alone. The government old age grant was the source of financial income for 240 members, and only 26 members perceived they had enough money to meet their financial responsibilities. Only a small fraction of the members used tobacco (7.3%) and alcohol (9.8%), whilst a more significant proportion ate fruit three times a week or more (89.8%) and vegetables daily (69.8%). The members claimed they were engaged in their communities – attending places of worship (84.7%), weddings (87.3%), funerals (51.5%), social clubs (85.1%), community meetings (57.1%) or visiting with family and friends (66.5%) and some did some voluntary work (24.7%). Most members (76.7%) felt they had an adequate social support system. The favourite individual activities the members engaged in were arts and crafts (98.2%), watching television (TV) (90.9%), helping neighbours (83.3%), walking (80.0%), reading (66.5%), doing voluntary work (24.0%), structured physical exercise (15.6%), farming (13.1%), sewing (2.5%), cooking (2.2%) and gardening (1.5%).

Table 3 and Table 4, respectively, list the self-reported health problems and members’ signs and symptoms. A total of 218 members were on some medication prescribed by self (*n* = 117), by doctors (*n* = 98), by nurses (*n* = 7) or by a herbal medicine practitioner (*n* = 1). Most of those on medication (205/218) claimed they remembered to take their medicines without any prompting.

**TABLE 3:** Self-reported health problems of members of service centers (*n* = 275).

Self-reported health problems†	*n*	%
Hypertension	163	59.3
Diabetes	43	15.6
Arthritis	29	10.5
Cataracts	24	8.7
Visual impairment	22	8.0
Asthma	16	5.8
Heart disease	14	5.1
Dizziness/fainting	12	4.4
Lower leg amputation	12	4.4
Blindness	8	2.9
Stroke	7	2.5
Osteoporosis	6	2.2
Falls	1	0.4

† , Some members reported multiple morbidities.

**TABLE 4:** Self-reported signs and symptoms of members of service centres (*n* = 275).

Signs and symptoms	Severity	*n*	%
Can you hear when someone talks to you?	Yes, hearing is good	207	75.3
	Yes, but not always	65	23.6
	No, never	2	0.7
Can you follow conversations when you are with several people?	Yes, I do	166	60.4
	Yes, but not always	80	29.1
	No, never	28	10.2
How much difficulty do you have in seeing and recognising an object or a person you know across the road?	None	134	48.7
	Mild	57	20.7
	Moderate	55	20.7
	Severe	21	7.6
	Extreme/cannot do	6	2.2
How much difficulty do you have in seeing and recognising an object at arm’s length, for example, reading or sewing?	None	138	50.2
	Mild	66	24.0
	Moderate	47	17.1
	Severe	11	4.0
	Extreme/cannot do	12	4.4
Do you lose control of your urine?	Yes	173	62.9
	No	95	34.5
Can you stand without support for 1 min?	Yes	263	95.6
	No	9	3.3
Can you walk without support for 10 min?	Yes	249	90.5
	No	22	8.0
Have you fallen within the past year?	Yes	90	32.7
	No	182	66.2
Are you afraid of falling?	Yes	213	77.5
	No	47	17.1

Members’ responses to the World Health Organization Disability Assessment Schedule (WHODAS) 2 questionnaire (Table 5) provided data relating to their health and disability in the six domains of cognition, mobility, self-care, getting along, life activities and participation. Significantly, few members expressed severe or extreme difficulty in these domains.

**TABLE 5:** Responses of members of centres to World Health Organisation disability assessment schedule 2 questionnaire.

Item		*n*	%
In the past 30 days how much difficulty did you have in:			
Standing for long periods such as 30 min?	None	165	60.0
	Mild	21	7.6
	Moderate	35	12.7
	Severe	36	13.1
	Extreme/cannot do	12	4.4
Taking care of your household responsibilities?	None	145	52.7
	Mild	38	13.8
	Moderate	59	21.5
	Severe	20	7.3
	Extreme/cannot do	5	1.8
Learning a new task, for example, learning how to get to a new place?	None	152	55.3
	Mild	44	16.0
	Moderate	59	21.5
	Severe	11	4.0
	Extreme/cannot do	3	1.1
How much of a problem did you have joining in community activities?	None	155	56.4
	Mild	41	14.9
	Moderate	46	16.7
	Severe	15	5.5
	Extreme/cannot do	12	4.4
How much have you been emotionally affected by your health problems?	None	133	48.4
	Mild	50	18.2
	Moderate	53	19.3
	Severe	32	11.6
	Extreme/cannot do	1	0.4
Concentrating on doing something for 10 min?	None	214	77.8
	Mild	28	10.2
	Moderate	18	6.5
	Severe	7	2.5
	Extreme/cannot do	2	0.7
Walking a long distance such as a kilometre or equivalent?	None	163	59.3
	Mild	28	10.2
	Moderate	38	13.8
	Severe	30	10.9
	Extreme/cannot do	8	2.9
Washing your whole body?	None	226	82.2
	Mild	18	6.5
	Moderate	16	5.8
	Severe	5	1.8
	Extreme/cannot do	4	1.5
Getting dressed?	None	234	85.1
	Mild	10	3.6
	Moderate	14	5.1
	Severe	6	2.2
	Extreme/cannot do	4	1.5
Dealing with people you do not know?	None	210	76.4
	Mild	29	10.5
	Moderate	13	4.7
	Severe	15	5.5
	Extreme/cannot do	1	0.4
Maintaining a friendship	None	200	72.7
	Mild	35	12.7
	Moderate	22	8.0
	Severe	11	4.0
	Extreme/cannot do	1	0.4
Your day-to-day work?	None	187	68.0
	Mild	30	10.9
	Moderate	39	14.2
	Severe	11	4.0
	Extreme/cannot do	1	0.4

Regarding their psychological health, members (*n* = 275) believed that their lives were meaningful (76.0%) and satisfied with themselves (84.0%), their sleep patterns (63.6%) and their energy needed for daily activities (65.5%) and with access to transport (*n* = 79.3%) and health services (68.7%). They were also satisfied with their living environments (69.8%), expressing a sense of safety (64.7%) in a healthy environment (62.9%), although 43% expressed feelings of despair, anxiety and depression. Concerning social interactions, most participants expressed satisfaction regarding their personal relationships with the people around them (66.9%) and the support they received from them (61%). Only 29% of the members reported satisfaction with their sex life.

## Discussion

In the absence of previous studies in the Eastern Cape province, the outcomes of our study provide preliminary baseline data on the characteristics of the service centres and their members in the province. Abdi et al. (2019) recommended that it is necessary, first, to understand older persons’ care and support needs, in order to develop effective solutions to address the needs identified in the preliminary data. Two issues stand out in the preliminary baseline data about the centres. These relate to the ability of the managers to manage the centres and the limited accessibility of healthcare services for the members.

Most of the members in the centres (*n* = 275) were women and able to carry out their daily activities. They engaged in healthy behaviours such as eating fruit and vegetables, reading, walking, exercising and interacting with their communities. Generally, the members reported that they had social interaction and participated in activities related to self-care, domestic life and mobility. They also perceived they had meaningful psychological health, hence satisfying the three areas of needs reported by Abdi et al. (2019). Only about 14% of the older persons had no formal education, and a similar small fraction of the members were engaged in risky health behaviour such as using tobacco and consuming alcohol. Over half of the members reported being hypertensive, having difficulties with sight and urinary incontinence and fearing falling, although whilst only one member had reported a fall, 33% said they had fallen within the past year. This discrepancy in the reporting of falls could have arisen if there were inconsistencies in the description of a fall provided by the assistants despite the prior training of the research assistants. The members also reported being emotionally affected by their health problems. Despite the prevailing challenges in the province, living within their communities, the members expressed general satisfaction with their lives, despite their long-term illnesses. This view is similar to the reports of Statistics South Africa (2020) and observations of Mayosi and Benatar (2014), relating to the country’s trends in increased life expectancy. It is important to note, however, that the profile of older persons in this study did not fit the general profile described by Ayodede et al. (2017), who reported a high prevalence of multi-morbidity associated with risk factors such as smoking, alcohol consumption and low levels of schooling. Unfortunately, despite the reported positive perception of wellness, the managers of the centres reported a lack of knowledge on how to care for older persons. It should also be observed that, with the challenges in accessing all the centres randomly selected for this study, it is possible that the centres that participated unintentionally attracted a biased sample of members on the day data collection took place, which excluded those who engaged in risk behaviours, had negative self-perceptions or manifested poor health.

Whilst updated information on the life expectancy at 60 years for the province was inaccessible, the impact of the long-term illnesses may negatively impact the average life expectancy at 60 years, as well as the quality of life, which could both be exacerbated by the minimal access to healthcare services provided in the centres. It was in only one service centre that members had access to nurses. The periodic screening and assessment of community-dwelling older persons are considered vital to detect the early onset of functional decline (Siemonsma et al. 2018). Unfortunately, less than 40% of the centres offered members screening opportunities for factors that could contribute to functional decline (e.g. monitoring vision, hearing, falls, cholesterol and blood pressure). However, the centres offered physical activity programmes that included walking, dancing and swimming although the frequency and level at which the members participated in the physical activities was not determined in our study. The possible benefits of the members’ participation in physical activity could have contributed to their ability to live independently and engage in their daily activities, reduced their risk of falling and improved the management of their reported morbidities, including hypertension, diabetes and arthritis. Therefore, the centres could have been proactive in contributing to the delay of functional decline through the physical activity programmes. To maximise the benefit of these services, interdisciplinary interaction between healthcare and non-healthcare professionals would be appropriate in the provision of the services in the centres. Such provision is acknowledged in the guidelines provided by the 2006 act for the training of healthcare providers in the care of older persons in South Africa (Phaswana-Mafuya et al. 2013; Ramklass et al. 2010).

When the services available in the centres were explored in relation to the six determinants of the framework (WHO 2002b), the health and social care systems determinants seemed to be a significant priority in the province although some of the services provided in the centres could also contribute to the social environment and behavioural determinants, as reported in similar studies in other countries (Arifin, Braun & Hogervorst 2012; Bélanger et al. 2017; Bowling 2006; Chansarn 2012; Fernández-Ballesteros et al. 2010; Paúl et al. 2012). The poor funding for the centres could have contributed to the paucity of evidence of activities targeting the remaining three determinants (personal, physical environment, economic) although it seemed that the centres provided platforms for assisting the older persons’ care in the community, which would contribute to ensuring their independent living, as guaranteed in the South African *Older Person’s Act* of 2006. Whilst the absence of healthcare professionals at the community level in the province has been reported (Ned et al. 2017), the availability of CHWs and their contribution to healthcare in the province has been observed (Austin-Evelyn et al. 2017; Laurenzi et al. 2021). In view of the numbers of the current generation of older persons in the province and the anticipated increase in the number of older persons when the NDP goal of increasing the average life expectancy to 70 years is achieved, it is necessary to incorporate the screening of older persons into the training of CHWs. The findings of Laurenzi et al. (2021) highlight the critical roles that CHWs could assume in providing both instructive and supportive care to clients and their informal caregivers whilst working under the supervision of healthcare professionals, including physiotherapists. However, based on the reported positive health behaviours of the members of the centres, their self-perception and engagement in physical and social activities, the philosophy of the training of the CHWs should shift from the deficit-approach of care to building on the strengths of older persons, using the principles of health promotion interventions in the care of older persons in their communities. For example, the instructive care should include the facilitation of exercise programmes for older persons, and physiotherapists should facilitate this training for CHWs (Amosun & Harris 2018). The training of CHWs should also include the care of clients with co-morbidities. The physiotherapists can also contribute to the training of the managers who requested more knowledge about how to manage older persons and their behavioural patterns.

Unfortunately, little attention is being given to the training of providers of care to healthy older persons (Tanyi & Pelser 2019), including by physiotherapists and CHWs. Despite consultations with officials at the provincial DSD, there was no evidence of a formal training programme to prepare the centres’ managers for their roles in planning to provide services that address the needs of older persons. Similar concerns have been raised as to whether the curriculum for the training of physiotherapists in South Africa is informed by the South African *Older Person’s Act* of 2006 (Amosun 2014; Ramklass et al. 2010). As physiotherapists are part of the interdisciplinary team whose focus should include the prevention or delay of the onset of functional decline, the curricula for the training of the different care providers (including CHWs) should include an awareness of the need for care and support, specifically in the areas of social activities and relationships, psychological health and activities related to mobility, self-care and domestic life (Abdi et al. 2019). A poor understanding of the needs of older persons would compromise the care models and support services provided to meet their needs (Abdi et al. 2019; Ayodede et al. 2017; He et al. 2012; Phaswana-Mafuya et al. 2013; Tuntland et al. 2015). Based on the reported needs of older persons who participated in our study, there is an opportunity to incorporate or strengthen physiotherapy services in the centres, using the principles of health promotion interventions. Collaborative care in addressing the needs of older persons requires that the curriculum of physiotherapy education exposes students to interprofessional teams so that they are able to share their physiotherapy skills with CHWs.

### Limitations

Caution needs to be exercised in generalising these province’s outcomes for two main reasons. Firstly, the service centres explored (*n* = 25) formed only 10.5% of the registered centres. The percentage of members surveyed was also a very small proportion of all the older persons in the province, that is, 2.2% of the service centres’ registered members and 0.05% of older persons in the population in 2017. These were similar to limitations reported in a similar survey of older persons in the Western Cape, where the number of service centres explored (*n* = 35) was 17.5% of the registered centres in the province (Amosun & Harris 2018). The participants in the Western Cape study (*n* = 625) also formed a very small proportion of older persons in the province, that is, 4.1% of the registered members of the service centres and 0.12% of older persons in the population in the 2011 census.

The reported motor vehicle accident brought the data collection to a stop, in addition to the difficulties in reaching the targeted centres when it rained. This limited data collection to only 28% of the selected centres. The change from randomised sampling to convenient sampling, because of challenges encountered in accessing the targeted service centres whenever it rained, added to the limitations in our study.

## Conclusion

Preliminary evidence suggests that the service centres for older persons in the Eastern Cape province provide platforms to fulfil some of the goals of the WHO’s active-ageing framework. Improving the health and social care determinants seems to be the priority although some of the services provided could also contribute to the social environment and behavioural determinants. Unfortunately, formal training for the centres’ managers was unavailable. Based on what was deduced about the contribution of physiotherapy to the care of older persons, the physiotherapy undergraduate curriculum should be reviewed. Finally, a comprehensive exploration of the members of the centres is needed as those who took part in our study could be a biased sample.
